# Effects of Domain-Specific Noise on Visual Motion Processing in Schizophrenia

**DOI:** 10.1371/journal.pone.0099031

**Published:** 2014-06-10

**Authors:** Yue Chen, Daniel Norton, Ryan McBain

**Affiliations:** 1 McLean Hospital, Department of Psychiatry, Harvard Medical School, Belmont, Massachusetts, United States of America; 2 Department of Psychology, Boston University, Boston, Massachusetts, United States of America; 3 Department of Global Health, Harvard School of Public Health, Boston, Massachusetts, United States of America; University of Akron, United States of America

## Abstract

**Background:**

Visual perception impairments in schizophrenia stem from abnormal information processing. Information processing requires neural response to a stimulus (signal) against a backdrop of 1) random variation in baseline neural activity (internal noise) and sometimes irrelevant environmental stimulation (external noise). Filtering out noise is a critical aspect of information processing, and needs to be critically examined in schizophrenia.

**Methods:**

To understand how noise in the visual system constrains perceptual processing, we devised a novel paradigm to build in both signal and external noise on same visual stimulus. Here, instead of uniformed noise, domain-specific noise—variations in stimulus speed—was introduced to evaluate the performance of schizophrenia patients in speed discrimination. Each motion stimulus—a random dot pattern (RDP) comprising 200 moving dots—included a range of speeds, drawn individually from a Gaussian distribution for each dot. The task for patients (n = 26) and controls (n = 28) was to identify which of two stimuli moved faster based on their mean speeds.

**Findings:**

Patients exhibited deficient speed discrimination at baseline, in the absence of speed noise. Their speed discrimination was further degraded in the presence of low and medium levels of external noise. In the presence of a high levels of noise, degradation of patients' speed discrimination leveled-off, resulting in similar performance to controls.

**Conclusion:**

These domain-specific noise effects on speed discrimination provide direct evidence for the existence of heightened internal noise within a specific visual motion processing domain in schizophrenia.

## Introduction

Schizophrenia has been considered as an information processing disorder in the brain [1], [2], [3], [4]. A central mechanism of information processing concerns how to disassociate signal from noise while dealing with relevant and irrelevant inputs. In the past decades, most perceptual and cognitive studies in schizophrenia patients have focused on the way various types of signals are encoded, which is one fundamental component of information processing. Many of these studies have found that patients fail to perform properly on perceptual, cognitive and motor tasks that rely upon external information [5], [6], [7], [8], [9], [10], [11], [12], [13], [14]. These findings have been largely attributed to poor signal encoding as a mechanism underlying perceptual processing in this mental disorder. In contrast, few studies have examined how noise affects information processing in schizophrenia.

Although poor filtering of noise has historically been hypothesized as a common thread among processing difficulties in schizophrenia [15], until recently the role of noise has not been considered with respect to the brain mechanisms implicated in this mental disorder. One framework, promoted by neurobiological studies, posits that the neural units and circuits for optimizing signal-to-noise ratio are a key factor in the pathophysiology of schizophrenia [16]. The notion that schizophrenia is associated with ‘noisy’ brain systems, as put forward in this framework, provides a theoretical basis for interpreting a cascade of information processing impairments in patients. Yet, direct evidence that points to the interference of noise in information processing, particularly at the perceptual and cognitive levels, has been lacking. The lack of empirical data leaves unanswered the question of whether and how noise in schizophrenic brains affects information processing independently from abnormal signal encoding.

Noise derives from two sources: The first is internal noise, arising from the random fluctuations of neural activity in the brain. The second source of noise is external, which arises from non-signal physical phenomena in the environment that affect neural and behavioral responses. In the sole presence of signals, internal noise in the brain exerts a limit on perceptual and cognitive capacities under various functioning conditions such as those in typical or aging adults [17], [18], [19]. Effects of the ever present internal noise are however by and large entangled with signal encoding in determining perceptual and cognitive responses. To disentangle the internal noise and signal encoding factors, one research strategy is to introduce signal-irrelevant physical stimuli or external noise. Responses to external noise are not directly related to signal encoding and can thus be used to evaluate the effects of internal noise on perceptual and cognitive processing.

Using external noise masking paradigms, two recent studies have suggested that the impaired perception of face and biological motion in schizophrenia patients may be attributed to an increased level of the internal noise [20], [21]. While these results support the notion of a “noisy brain” in schizophrenia, a common issue was that the external noise was introduced in addition to the stimuli carrying visual signals, and might thus evoke different perceptual and cognitive processes. For example, in the study by Kim and colleagues (2013)[21], the motion signals composing biological motion were juxtaposed with other dot motions which made seeing the motion of the figure more difficult. By adding additional stimuli to serve as noise, other visual and cognitive processes than the visual process of interest might be evoked. Evoking other visual and cognitive processes would create a confound as it became unclear whether the increased level of noise in schizophrenia was a general effect across visual and cognitive domains or was specific to the domain in which particular visual signals (such as movement speed) were processed. Distinguishing the two scenarios may not only illuminate the underlying mechanisms for visual processing impairments but also provide cues for designing visual interventions targeting noise reduction in this mental disorder.

In this study, we devised a novel paradigm to examine the effect of domain-specific external noise on visual motion perception in schizophrenia. Domain-specific noise was introduced by embedding both signal and noise within the same visual motion stimuli (i.e. no additional visual stimuli involved). This novel study-design allowed us to empirically evaluate whether the altered internal noise is intrinsic to a specific information processing system. The goal here was to evaluate the consequences of external noise on a specific visual process in schizophrenia, without evoking other visual and cognitive processes. Our working hypothesis was that, compared with controls, patients' performance on motion perception would be affected by the domain-specific external noise. This external-noise-induced effect would be less in patients than in controls, assuming that visual motion processing system in schizophrenia is already internally noisy.

## Methods

### Subject

Participants included 26 schizophrenia patients and 28 normal controls. These individuals were included based on the following criteria: 1) no history of any neurological disorders (such as seizure or stroke) or head injuries, 2) IQ>70, 3) age between 18 and 60 years old, and (4) no substance abuse in the six months prior to participation.

Patients were recruited from McLean Hospital and the Greater Boston areas. Their diagnoses were established based on a structured clinical interview SCID-IV [22] conducted by trained clinicians who were blind to the purposes of this study, and by a review of all available medical records. Thirteen of these patients had a diagnosis of schizophrenia and the rest had a diagnosis of schizoaffective disorder. All patients were medicated on antipsychotic drugs (mean CPZ = 538.0 mg, SD = 422.7 mg) [23]. The Positive and Negative Syndrome Scale [24] was administered to the patients (positive subscale = 14.0, SD = 6.9; negative subscale = 10.8, SD = 3.0; general subscale = 24.9, SD = 6.8). Healthy controls were recruited from the local community, and were screened to ensure the absence of Axis I psychiatric disorders using a standardized interview based on SCID-I/NP [25]. The two groups of subjects were matched in terms of age and gender composition.

The Wechsler Adult Intelligence Scale - Revised (verbal component) [26] was administered to all participants. The participants had normal or corrected to normal vision, as assessed by the Rosenbaum Pocket Vision Screener. Table 1 provides demographic information of the participants.

**Table 1:** Demographic Characteristics of the Sample.

	Sex	Age (year)	Verbal IQ*	Education (year)	Parental Education
Control (n = 28)	13 M, 15 F	43.0 (15.2)	111.5 (12.7)	15.3 (1.8)	14.7 (3.7)
Patients (n = 26)	16 M, 10 F	43.0 (9.5)	101.3 (11.1)	14.0 (2.0)	14.4(3.0)

Group Mean (Standard Deviation).

F- female; M- male.

*based on The Wechsler Adult Intelligence Scale.

### Stimulus

The visual motion stimulus was a random dot pattern (RDP) comprising 200 dots moving left or right. The speeds of the dots were dictated by a signal speed multiplied by a Gaussian distribution of varying standard deviations. The greater the standard deviation, the greater the noise level of the stimulus. The dots were small (2×2 min arc) and white, and presented on an otherwise black background. Spatial location of the dots was randomly distributed within a circular window (10 degrees of visual angle). Display time of each RDP was 300 msec.

**Figure 1:**
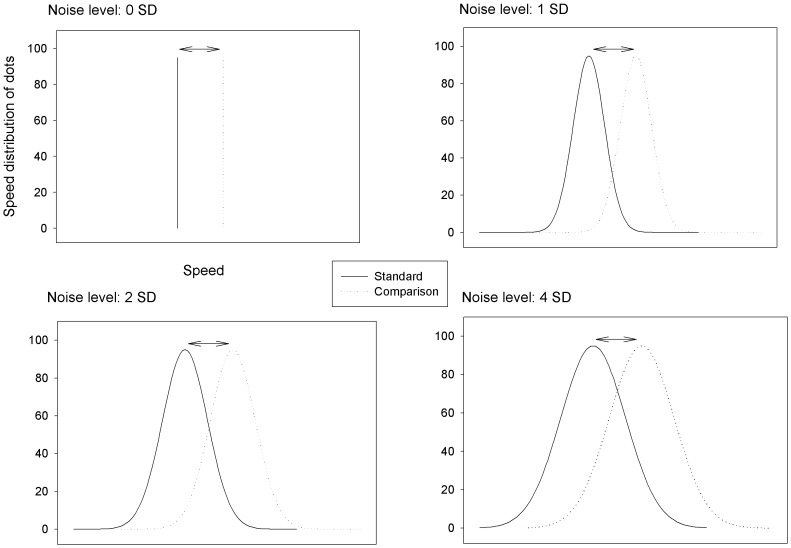
Schematic illustration of Gaussian speed profiles of the random dot patterns (RDPs) used for speed discrimination. Each panel corresponds to a pair of RDPs with Gaussian speed distribution of a specific bandwidth, i.e. a specific level of speed noise. The x axis represents the range of speed. The y axis represents relative distributions across speed (The values are arbitrary). Corresponding to no noise, 0 SD means that a single speed is used for all dots in an RDP. Corresponding to low, medium and high level noise, 1, 2 and 4 SD mean that the movement of each dot in an RDP was independently drawn from a Gaussian distribution of speed, with the bandwidths 2, 4, and 8 degrees/sec. For a certain speed difference (or Weber speed ratio), the wider the Gaussian distribution, the more difficult speed discrimination is.

Unlike in conventional RDPs, the speed of each dot in the RDP used here was drawn independently from a Gaussian distribution (Figure 1). The mean of the standard Gaussian speed distribution was 5 degrees/sec. The means of the comparison Gaussian speed distributions were 5.25, 5.5, 6, 7, 9 and 13.0 degrees/sec. The mean differences between the standard and the comparison Gaussian speed distributions generated six levels of signal strength for speed discrimination. The half-peak widths of the Gaussian speed distributions (SD) were 0 (uniform speed for an RDP), 1, 2, or 4 degrees/sec, generating four levels of speed noise (no noise (0 SD), low noise (1 SD), medium noise (2 SD) and high noise (4 SD)).

### Procedures

The task was to discriminate between a pair of RDPs, based upon the mean speed of each stimulus. Each trial included two presentations. One presentation contained an RDP in which the movement of dots had the standard Gaussian speed distribution (mean speed: 5.0 degrees/sec). The other presentation contained an RDP in which the movement of dots had one of the comparison Gaussian speed distributions (mean speed: 5.25, 5.5, 6.0, 7.0, 9.0 or 13.0 degrees/sec for the no-noise condition, and 5.5, 6.0, 7.0, 9.0, 13.0 or 21.0 degrees/sec for the low noise, medium noise and high noise conditions). These speed differences correspond to Weber ratios of 0.05, 0.1, 0.2, 0.4, 0.8, and 1.6 for the no noise conditions, and 0.1, 0.2, 0.4, 0.8, 1.6 and 3.2 for the noise conditions. The larger the speed difference between the standard and the comparison, the easier the speed discrimination would be. Because speed discrimination would be more difficult under the noise conditions, slightly larger speed differences were applied. Subjects determined which of the two presentations contained a faster moving RDP. This two-alternative forced choice procedure was administered with and without the presence of various levels of Gaussian speed noise. The four testing sessions were blocked according to the speed noise level (0, 1, 2, or 4 SD). With six levels of speed comparisons, two directions of motion (left and right) and each condition being repeated 8 times, each of the four sessions contained 96 trials.

The percent of correct trials or performance accuracy was used as a primary measure of visual performance. Only the accuracy data obtained under the identical Weber ratios (0.1, 0.2, 0.4, 0.8 and 1.6) for the no-noise and noise conditions were entered for analysis. The data under the Weber ratio of 0.05 (for the no-noise condition) and 3.2 (for the noise conditions) were acquired here to help derive perceptual thresholds under the respective conditions (see below).

The perceptual threshold was also used as a measure of performance that allowed direct comparison across noise levels. Thresholds were defined as the minimum signal strength level at which subjects perform at the 80% accuracy level [27].

All stimuli and task procedures were programmed in C on a G3 Mac computer, which also recorded subjects' responses. Subjects received instructions and practice time prior to formal data collection.

The study protocol was approved by the institutional review board (IRB) of McLean Hospital. Written informed consent was obtained from all the participants. Prior to that, patients' ability to consent was established through the endorsement of participating research study by treating psychiatrists during a medical record retrieval process and through a screening interview in which basic demographic information was collected. There was no surrogate consent procedure.

## Results

### Speed discrimination in presence of Gaussian speed noise

**Figure 2:**
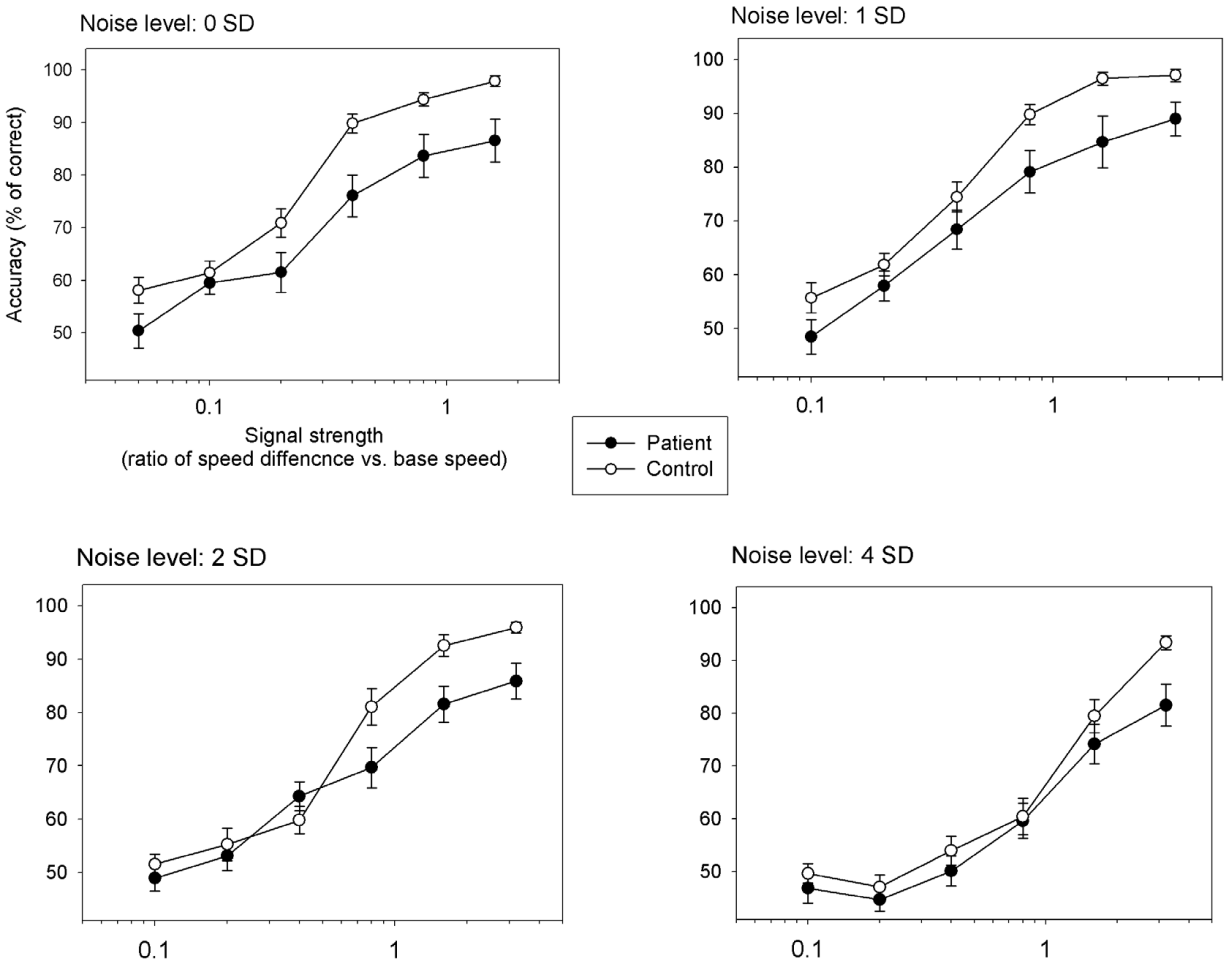
Group comparison of accuracies of speed discrimination as a function of speed noise level. In each panel, the x-axis represents Weber speed ratios [(standard speed – comparison speed)/standard speed] for a pair of random dot patterns, or the signal strength, used for speed discrimination. The y-axis represents the percent of trials in which a correct response is produced. Error bars indicate ±1 standard error.

A three-way ANOVA (2 groups×5 signal strengths×4 noise levels) of performance accuracy revealed significant effects on signal strength (F = 176.2, p<0.001), group (F = 41.2, p<0.001) and noise (F = 98.2, p<0.001). Significant interactions were found between signal strength and noise (F = 3.9, p<0.001), between group and noise (F = 3.3, p = 0.02), and between signal strength and group (F = 2.5, p = 0.04). The interaction among signal strength, group and noise was not significant (F = 1.3, p = 0.22). This overall analysis indicates that the group differences depend upon the level of noise (Figure 2). Additional analysis for each noise level then followed.

For each speed noise condition, a two-way ANOVA (group×signal strength) revealed significant effects on signal strength (no-noise: F = 50.0, p<0.001; low-noise: F = 60.4, p<0.001; medium-noise: F = 41.7, p<0.001; high-noise: F = 35.7, p<0.001) and on group (no-noise: F = 28.4, p<0.001; low-noise: F = 18.7, p<0.001). For the medium and high noise conditions, group effects were not significant. This analysis indicates significant group differences at no-noise and low-noise levels.

Only for the medium-noise condition, the interaction between group and signal strength was significant (F = 3.9, p = 0.004), indicating the existence of group difference that was dependent upon signal strength (Figure 2). Post hoc t tests showed performance levels of patients were significantly lower for the high, but not for the low, levels of signal strength at this noise level (Weber speed difference of 0.1: t = 0.94, p = 0.35; 0.2: t = 0.13, p = 0.90; 0.4: t = 0.62, p = 0.54; 0.8: t = 2.38, p = 0.02; 1.6: t = 2.39, p = 0.02). This indicates that for the medium-noise condition, the groups significantly differed at high levels of signal strength.

**Figure 3:**
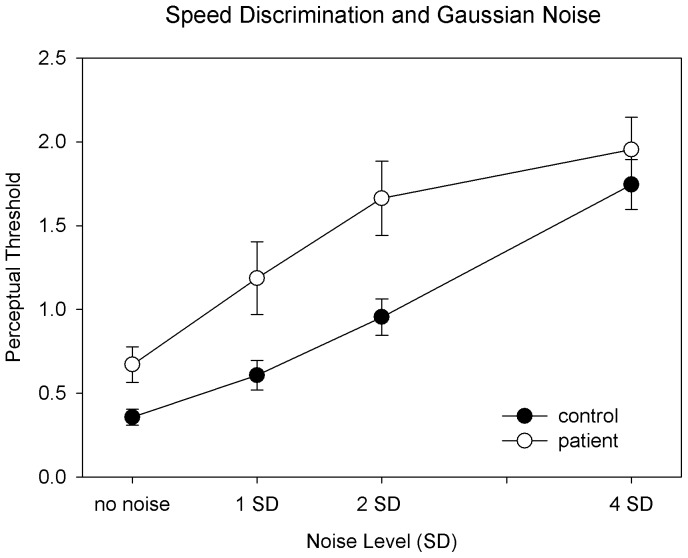
Comparisons of speed discrimination thresholds as a function of speed noise level. The x-axis represents the noise level (including the no noise condition) for a pair of random dot patterns used for speed discrimination. The y-axis represents the perceptual thresholds of speed discrimination. The lower a threshold, the better the perceptual performance is. Error bars indicate ±1 standard error.

A two-way ANOVA (group×noise) of perceptual thresholds revealed significant effects on group (F = 10.9, p<0.001) and noise (F = 17.3, p<0.001). The interaction effect was not significant (F = 0.63, p = 0.34). Post hoc t tests showed that the perceptual thresholds of patients were significantly elevated (lower performance level) for the conditions of no-noise (t = 2.68, p = 0.009), low-noise (t = 2.61, p = 0.01), and medium noise (t = 2.93, p = 0.005), but not for the condition of high noise (t = 1.06, p = 0.29) (Figure 3).

### Relationship with clinical variables

Perceptual thresholds for each speed noise condition were used as a unified performance measure to compare with clinical variables. Patients' perceptual thresholds were not significantly correlated with their PANSS scores (positive, negative or general), except for the one between the perceptual threshold under no speed noise condition and the positive PANSS score (r = 0.57, p<0.05). The correlations between the perceptual thresholds and illness duration or CPZ levels were also not significant.

## Discussion

This study found differential effects of motion domain-specific external noise on motion perception between patients and controls. Under the no noise condition, speed discrimination of the patients was degraded and similar to speed discrimination of the controls under the low-level noise condition. The presence of low- and medium-level noise significantly degraded speed discrimination of the patients, including under the condition where strong speed signals were available. However, with a further increase in the level of noise, the degradation of speed discrimination in the patients leveled-off, resulting in performance levels similar to controls.

In principle, both disrupted signal encoding and increased internal noise can cause impaired visual processing in schizophrenia. Yet, the relative roles of the two components are poorly understood. Through a parametric manipulation of domain-specific noise, this study provided a way to demonstrate visual processing performance as a function of noise level. The noise-induced performance degradations in patients showed three aspects of the role of noise in visual motion processing. First, the additive effects from the no external noise to the low-level external noise (1 SD) are consistent with the notion that patients' degraded speed discrimination results from heightened internal noise. If weakened signal, rather than heightened internal noise, were a major factor for the degraded performance in patients, the group difference would decrease with increase of signal strength. This was however not the case - the group difference was same or slightly enlarged for the high-level speed signals under the 1 SD condition (Figure 2), suggesting the existence of heightened internal noise in schizophrenia. Second, with the increase of the external noise level (2 SD), patients' performance was degraded to a greater extent for high-level speed signals, suggesting that the combination of heightened internal and external noise outweighs even strong motion signals during the processing of visual motion information. Third, a further increase of external noise (4 SD) made internal noise, heightened or not, relatively less salient and imposed a major drive that degraded controls' and patients' performance. This effectively led to similarly degraded performances in the two groups. These three aspects of the results (Figure 2) highlight heightened internal noise as a primary factor limiting visual performance under the low and medium level external noise conditions in schizophrenia.

**Figure 4:**
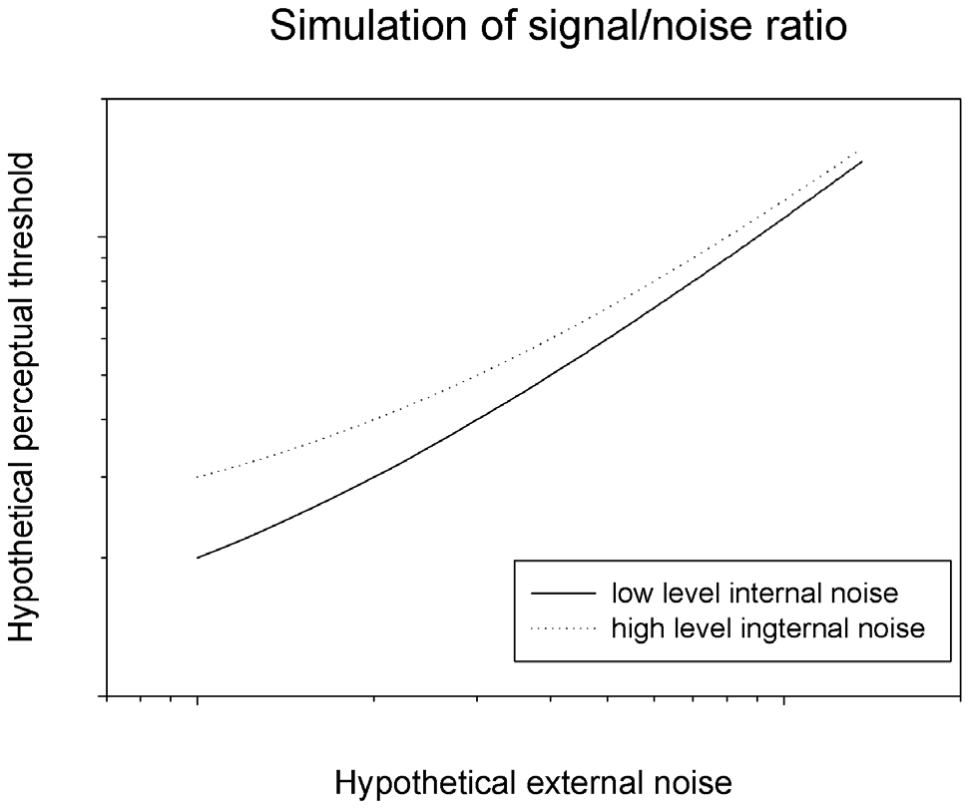
Simulated perceptual threshold based upon a signal/noise ratio model. The signal/noise ratio model assumes that perceptual sensitivity is proportional to a ratio of signal strength and noise (internal and external). Or, in this case, hypothetical perceptual threshold (an inverse of perceptual sensitivity) ∝ (internal noise + speed noise)/speed difference. By inputting a low and a high level internal noise, this formula produces two types of hypothetical perceptual threshold as a function of external noise that resemble empirically acquired perceptual thresholds of controls and patients, respectively (Figure 3).

These characteristics of the results can be captured by a basic information processing model in which perceptual performance is principally constrained by a signal/noise ratio [perceptual sensitivity ∝ signal (speed difference)/(internal noise + external noise (speed noise)) (‘∝’ signifies proportional) or hypothetical perceptual threshold (an inverse of perceptual sensitivity) ∝ (internal noise + speed noise)/speed difference, in this case]. This model predicts differential effects of external noise on performance in the presence of low level (controls) vs. high level (patients) internal noise (Figure 4). This prediction mirrors the empirical findings of the present study (Figure 3).

Several previous studies have shown that the presence of external noise alters perceptual responses in schizophrenia. On tasks which assess the perception of coherent motion, a group of dots moving in random directions (i.e. direction noise) was often included alongside the target, another group of dots moving coherently in one direction [28]. To perform the motion perception task, patients required a larger proportion of signal dots or a smaller proportion of noise dots [29], [30], suggesting that either heightened internal noise or weakened signal encoding undermines their performance levels. In perceiving biological motion and face perception, when target stimuli were combined with noise stimuli the degradation of patients' performance depended upon the noise level, a result consistent with the existence of heightened internal noise [20], [21]. In visual and auditory speech perception, when various levels of non-uniform and frequency-dependent noise were present patients showed deficits specifically under the condition where sensory integration was optimal for controls [31]. By showing that external noise, in addition to signal, differentially affected patients' perceptual performance, these results are consistent with the notion that internal noise contributes to impaired perceptual processing in this mental disorder. On the other hand, the added noise employed in these previous studies could become another source of sensory signals that evokes additional perceptual and cognitive processes. Thus, the roles of signal and noise in visual performance remained entangled in schizophrenia.

The novel design of this study embeds signal and noise within the same visual stimuli – with each dot carrying information about speed and speed noise. This domain-specific noise does not evoke additional perceptual processes, thus allowing the role of noise to be characterized within the specific visual motion processing domain. The pattern of noise-induced performance degradation in patients suggests the existence of heightened internal noise in the visual motion processing system.

Another study on beauty perception found that adding uniform noise to original artwork produced similar effects on the beauty rankings and ratings in patients and controls [32]. The lack of non-domain specific noise effect seems to support the notion that the type of external noise utilized such as domain-specific vs. non-domain-specific is critical in determining its impact on information processing impairments in schizophrenia.

Heightened internal noise in schizophrenia likely stems from abnormal brain activity. It has been reported that patients' activations in multiple cortical regions, such as hippocampal, thalamic and temporal areas, are abnormally increased in the presence of noisy auditory stimuli [33], [34]. It has been shown that increased variability in brain responses is associated with decreased variability in behavioral performance of healthy people [35]. Whether noisy schizophrenic brains are better characterized by increased response amplitude or by altered response variability remains an open question. How patients' abnormal cortical and perceptual responses are linked in the context of domain-specific noise vs. non-domain-specific noise is a topic that merits further investigation.

This study found that low to medium level external domain-specific noise imposes an additive effect in degrading visual performance in patients. One implication of this susceptibility to domain-specific noise is that, for stimulus-based behavioral interventions to be effective, the design should consider providing increased saliency of visual presentations (i.e., an increase of signal-noise ratios), explicit instructions on perceptual procedures and direct feedbacks on perceptual responses. Recently, it has been shown that variability in brain response (i.e. internal noise) can be modified by behavioral tasks [36]. This result suggests a possibility of reducing heightened internal noise in a specific brain system (such as in the visual processing domain) through targeted behavioral interventions. Such a ‘de-noise’ approach should be particularly helpful for patients to focus on relevant visual information, and minimize domain-specific distractions, as irrelevant information may be automatically filtered out by healthy people.

Through the use of domain-specific external noise, this study found that the internal noise within the visual motion system is heightened in schizophrenia, and contributes to patients' impaired performance on visual motion perception. Future studies should further differentiate domain-specific from non-specific noises in visual information processing and specify the roles of noise in different domains of information processing in schizophrenia.
